# The Local Origin of the Tibetan Pig and Additional Insights into the Origin of Asian Pigs

**DOI:** 10.1371/journal.pone.0028215

**Published:** 2011-12-07

**Authors:** Shuli Yang, Hao Zhang, Huaming Mao, Dawei Yan, Shaoxiong Lu, Linsheng Lian, Guiying Zhao, Yulin Yan, Weidong Deng, Xianwei Shi, Shuxin Han, Shuai Li, Xiujuan Wang, Xiao Gou

**Affiliations:** 1 College of Animal Science and Technology, Yunnan Agricultural University, Kunming, China; 2 College of Animal Science and Technology, China Agricultural University, Beijing, China; The University of Queensland, St. Lucia, Australia

## Abstract

**Background:**

The domestic pig currently indigenous to the Tibetan highlands is supposed to have been introduced during a continuous period of colonization by the ancestors of modern Tibetans. However, there is no direct genetic evidence of either the local origin or exotic migration of the Tibetan pig.

**Methods and Findings:**

We analyzed mtDNA hypervariable segment I (HVI) variation of 218 individuals from seven Tibetan pig populations and 1,737 reported mtDNA sequences from domestic pigs and wild boars across Asia. The Bayesian consensus tree revealed a main haplogroup M and twelve minor haplogroups, which suggested a large number of small scale *in situ* domestication episodes. In particular, haplogroups D1 and D6 represented two highly divergent lineages in the Tibetan highlands and Island Southeastern Asia, respectively. Network analysis of haplogroup M further revealed one main subhaplogroup M1 and two minor subhaplogroups M2 and M3. Intriguingly, M2 was mainly distributed in Southeastern Asia, suggesting for a local origin. Similar with haplogroup D6, M3 was mainly restricted in Island Southeastern Asia. This pattern suggested that Island Southeastern Asia, but not Southeastern Asia, might be the center of domestication of the so-called Pacific clade (M3 and D6 here) described in previous studies. Diversity gradient analysis of major subhaplogroup M1 suggested three local origins in Southeastern Asia, the middle and downstream regions of the Yangtze River, and the Tibetan highlands, respectively.

**Conclusions:**

We identified two new origin centers for domestic pigs in the Tibetan highlands and in the Island Southeastern Asian region.

## Introduction

Tibetan pigs are mainly distributed in the Tibetan highlands, the largest continuous high elevation ecosystem in the world with an average elevation of more than 4000 m. These animals show striking phenotypic and physiological differences from lowland pigs that have allowed them to adapt to the extreme conditions such as hypoxia. In addition, domestic pig bones discovered in the Tibet archaeological site, Kanuo, that are estimated to be around 5,000 years old indicate that Tibetan people could domesticate the Tibetan pig [1]. This discovery also supports that the Tibetan pig may have a local origin. However, there is no direct genetic evidence of either the local origin or exotic migration of these animals. Generally, the domestic animals currently indigenous to the Tibetan highlands are, with the exception of the yak, believed to have been introduced during a continuous period of colonization by the ancestors of modern Tibetans. The earliest evidence of human activity and yak husbandry (Qiang Culture) has been found in this region,suggesting that wild yaks (*Bos grunniens*) were first domesticated in Tibet [2]. Previous studies using mitochondrial DNA (mtDNA) also suggest that Tibetan yak have a local origin [3]. Therefore, to use a comparative mtDNA analysis of the Tibetan pig with relevant populations across Asia we may be able to elucidate the origin of this breed and the degree and pattern of introgression of genetic material from exotic breeds into Tibetan pigs.

The rough genetic relationship between wild boars and domestic pigs has been studied by comparing the mtDNA control region between a relatively small sample of animals [4]–[10]. These earlier reports demonstrate that Asian-type pigs were only recently diverged from each other, whereas they indeed have significant differences from European-type breeds. Furthermore, studies using nuclear DNA markers have also documented a distinct genetic distance between the European and Asian pig populations [11]–[13]. Recently, comprehensive studies have been performed and showed a worldwide phylogeography of wild boars and domestic pigs [14]–[19]. The first systematic study [14] on mtDNA polymorphisms in wild boar and domestic pig populations covering almost the entire globe revealed that pigs were domesticated in at least six different centers. Further studies using appropriately sampled populations in the Island Southeastern Asian [15], Pacific Islands [17], Southern Asian [19], Southeastern Asian [18], [19] and Eastern Asian [18] regions have proposed that the Pacific clade of pigs was recently domesticated within Southeast Asia. Furthermore, Asian pig domestication appears to have occurred mainly in Northeastern India, the Mekong region, and the middle and downstream regions of the Yangtze River. Interestingly, the three centers of domestication are all near the Tibetan highlands.

The specific genetic effects of the Asian centers of origin (listed above) on the Tibetan pig are unclear. In this study, the commonly used hypervariable fragment (HVI) in the mtDNA control region was used to characterize domestic pig and wild boar diversity across a wide range of pig breeds. In addition to 1,714 sequences retrieved from GenBank, 241 novel individuals were sampled from different regions where the samples have not been adequately collected before. These regions contained 12 pig populations indigenous to Yunnan province and the Tibetan highlands of China. Therefore, we here provide the most comprehensive screening of mtDNA variations among domestic pigs and wild boars in Asia to obtain genetic information on the evolutionary domestication history of the Tibetan pig.

## Materials and Methods

### Sampling and sequencing

**Figure 1:**
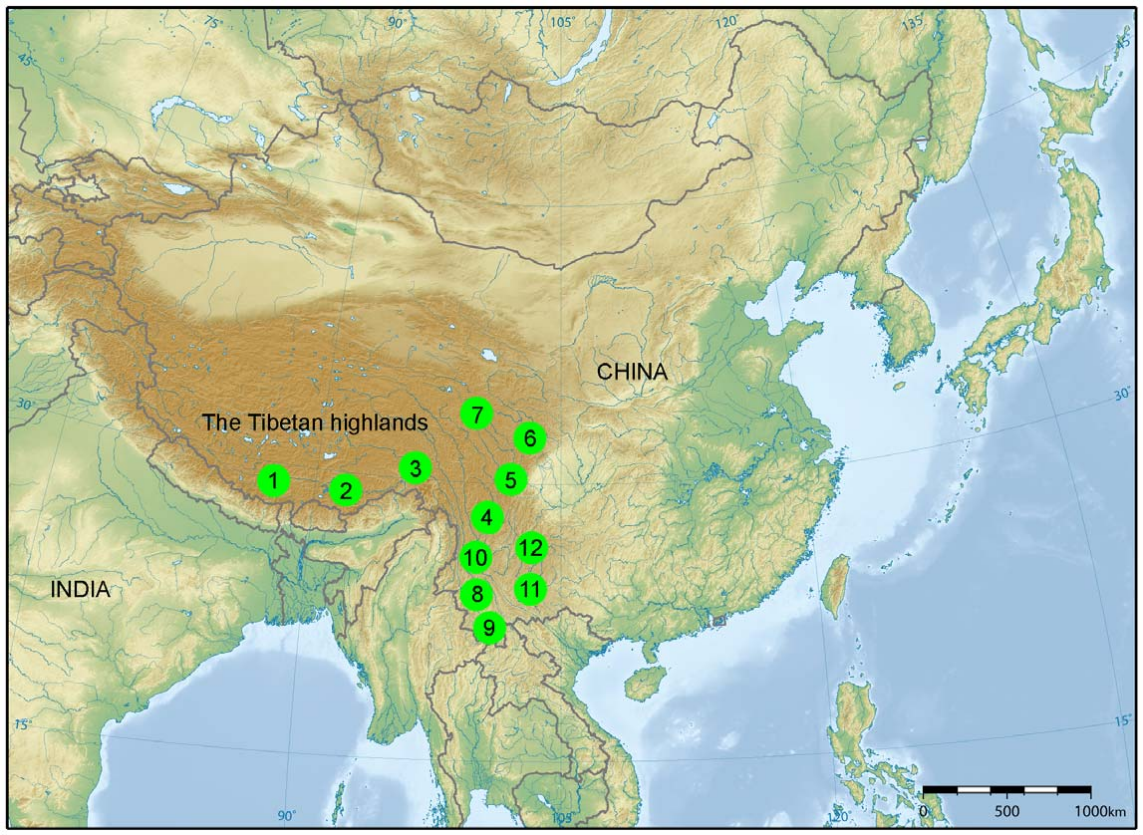
Sampling locations used in the study. Codes 1–7 represent the Tibetan pig populations of Shannan, Linzhi, Changdu, Diqing, Ganzi, Aba and Hezuo, respectively. Codes 8–12 represent the Yunnan pig populations of Baoshan, Diannanxiaoer, Gaoligongshan, Saba and Wujin, respectively.

Prior approval by the Institutional Animal Care and Use Committee of Yunnan Agricultural University (Contract 2007-0081) was given for all experimental procedures involving animals in the present study. Blood or tissue samples of 241 individuals were collected from pigs indigenous to Yunnan province (n = 102) and the Tibetan highlands (n = 139) of China (Fig. 1). The animals sampled were the Yunnan native pigs (Diannanxiaoer n = 26, Baoshan n = 19, Saba n = 41, Gaoligongshan n = 10, Wujin n = 6), and the Tibetan pigs (Linzhi n = 23, Shannan n = 11, Changdu n = 17, Diqing n = 39, Aba n = 12, Ganzi n = 10, and Hezuo n = 27). DNA was isolated using standard SDS/proteinase K extraction methodology or the Qiagen blood and tissue extraction kit (Qiagen, Valencia, USA). Based on the complete pig mtDNA sequence [20], a primer set (5′-^15336^CCCAAAGCTGAAATTCTAAA-3′, 5′-^15822^GGTGAGATGGCCCTGAAGTAAG-3′) was designed to amplify a 431 bp segment of the commonly used mtDNA control region (HVI). PCR products were sequenced directly using the ABI PRISM Dye Terminator Cycle Sequencing Ready Reaction Kit (Applied Biosystems) and electrophoresed on an ABI 3700 PRISM DNA sequencer (Applied Biosystems). All 241 sequences were submitted to GenBank, with reference numbers HQ148311–HQ148551.

### Data analysis

To obtain good coverage of pig breeds, the dataset of 241 samples was complemented with data on 1663 domestic pigs and 292 wild boars. A list of all samples, with GenBank accession number, breed and geographical origin, is provided in an editable format in Supplementary Table S1. The sequences were edited and aligned by the DNAstar package (DNASTAR, Madison, WI), and the polymorphisms in the analyzed segments were exported using MEGA3 [21]. The African warthog (*Phacochoerus africanus*) is known to be distinct from the Eurasian wild boars and was consequently used as an outgroup for the phylogenetic analyses. Gaps in the aligned sequences were excluded from subsequent analysis. Bayesian consensus (MB) trees were constructed using the Monte Carlo Markov chain (MCMC) approach by Bayes 3.1 [22]. Four independent runs were performed, each with 5 million generations. The posterior probabilities listed on the trees represent the lowest recorded values between all of the runs. The median joining network plots of mtDNA control region sequences [23] were constructed using the Network4.1 program (http://www.fluxus-engineering.com/sharenet.htm).

To test the relationship between the Tibetan pig and other lowland breeds, the distribution of pigs in Asia was partitioned into the 15 geographical regions referred to in previous studies [14], [18]: the Tibetan highlands, South Asia (SA), Yunnan, South East Asia (SEA), Island South East Asia (ISEA), Pacific Islands, the upstream region of the Yangtze River (URYZ), the middle and downstream region of the Yangtze River (MDYZ), South China (SC), Taiwan, the middle and upstream region of the Yellow River (MUYR), the downstream region of the Yellow River (DRYR), North East China (NEC), Japan and Korea.

## Results

### Sequence variations and haplotypes

We sequenced the mtDNA control region of 241 novel pig samples in the present study, and also downloaded 1,714 Asian pig sequences from GenBank. In total, 229 haplotypes were found using data (Table S1) from this sample of 1,663 domestic pigs and 292 wild boars (Table S2). Of these haplotypes, 162 existed only in domestic pigs, 85 existed only in wild boars, and 18 were shared between both (Table 1). Forty-three haplotypes were identified in the 218 individuals from the seven Tibetan pig populations. The number of haplotypes present in animals from the Tibetan highlands was higher than in animals from any other region (Table S2).

**Table 1:** Haplotype distribution between domestic pigs and wild boars.

Haplogroup	No. ofhaplotypes		No. ofIndividuals		Region
	Domesticpig	Wildboar	Domesticpig	Wildboar	
M	112	44	1497	191	Throughout Asia
D1	15	-	17	-	Tibetan highlands
D2	2	-	8	-	URYR and MDYZ
D3	2	-	3	-	Tibetan highlandsand Yunnan
D4	3	-	5	-	SA
D5	2	-	13	-	SEA
D6	2	-	13	-	ISEA andPacificIslands
D7	4	-	16	-	Yunnan,Tibetan highlands,SEA andISEA
D8	5	-	42	-	MDYZ,MUYRand SC
DW1	4	2	12	10	SA
DW2	1	1	1	1	Taiwan
DW3	1	1	1	2	SEA
W1	-	5	-	8	SA
W2	-	2	-	6	Korea
W3	-	3	-	3	Japan
W4	-	2	-	2	Japan
W5	-	2	-	2	SEA
W6	-	6	-	20	URYZ,MDYZ,MUYRand SC
W7	-	16	-	41	SEA, Taiwanand Korea
E	9	1	31	1	ISEA, SA, MDYZ,Korea and Japan

Short lines denote that no samples were collected, and the abbreviations for the regions are explained in the main text. Haplogroups are referred to in Figure S1.

### Phylogenetic analysis of domestic pigs and wild boars in Asia

The tree topology for the 229 haplotypes obtained using the Bayesian (MCMC) phylogenetic method is presented in Figure S1. This Bayesian consensus tree revealed 20 divergent haplogroups, of which eight (labeled D1 through to D8) were found only in domestic specimens, seven (labeled from W1 to W7) were found only in wild boars, four (labeled M, DW1, DW2 and DW3) constituted a mix of wild and domestic samples, and the remaining one haplogroup (termed E) belonged to a European cluster (Figure S1 and Table 1). Most of haplogroups have been defined by high posterior probabilities (above 70%), implying reliable structure among these haplogroups.

The main haplogroup M is the most frequently represented group in Asian breeds with respect to both the number of samples and haplotypes (Tables 1 and S2). Among the 229 haplotypes found in the total sample of animals used in the present study, haplogroup M was represented by 141 haplotypes found in 1,497 domestic pigs and 191 wild boars. By contrast, the 19 minor haplogroups harbored only 88 haplotypes which were found in 166 domestic pigs and 101 wild boars (Tables 1 and S2). However, the D1 individuals were restricted to 15 haplotypes unique to the Hezuo pig of the Tibetan highlands. In addition, the DW1 is an independent group shared between domestic pigs and wild boars from SA, including India, Bhutan and Sri Lanka. Yet, D4 is another independent SA group that exists in Bhutanese pigs. The groups D5 and D7 were mainly found to be distributed in SEA. Interestingly, group D5 is restricted only to Cambodia and Laos, whereas group D7 is found mainly in Myanmar and Thailand. Likewise, groups DW2 and DW3 are also shared between domestic pigs and wild boars in Taiwan and Thailand respectively, whereas group D6 is mainly located in the Pacific Islands and ISEA region. By contrast, groups D2, D3 and D8 are dispersed throughout China. These 12 groups are regionally distributed despite the existence of only a small number of individuals and haplotypes, which suggests that a large number of small scale *in situ* domestication episodes occurred. Group E animals likely originated from European breeds as this group harbors 9 European haplotypes (representing 32 samples), and they are mainly in the regions of ISEA, SA, MDYZ, Korea and Japan. In addition, the seven wild boar groups also exhibit regional distributions, including one SA group (W1), two Korean groups (W2 and W7), two Japanese groups (W3 and W4), one SEA group (W5), and one Chinese group (W6).

### Network analysis of Haplogroup M

**Figure 2:**
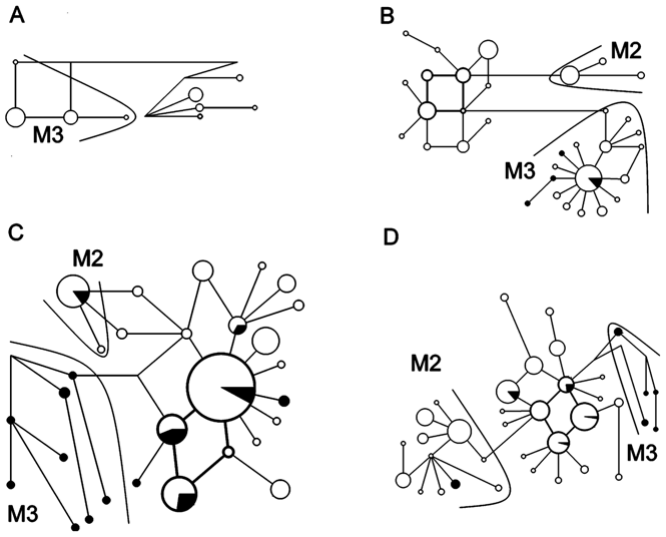
Median joining networks of haplogroup M in the Pacific Islands (A), ISEA (B), Yunnan (C) and SEA (D) regions. The haplotypes are symbolized by circles and separated by one substitutional step. The length of each branch is proportional to the number of associated mutations. The size of the circle is proportional to the frequency of the haplotype and the white and black areas of the circle represent the proportion of this frequency made up by domestic pigs and wild boars respectively. Subgroup M2 is identified in B, C and D, and subgroup M3 in A, B, C and D.

Haplogroup M harbors three subgroups M1, M2 and M3. Only the regions containing subgroup M2 or M3 are shown in Figure 2 (the Pacific Islands in A, ISEA in B, Yunnan in C, and SEA in D). Nevertheless, the subgroup M2 in Figure 2*D* and M3 in Figure 2*B* exhibit a star-like distribution profile that is typical of exponential population growth. Each of them harbors core haplotypes that have a series of one, two, or greater than two mutation distance derivatives (derived haplotypes) detected in domestic pigs and wild boars. Therefore, taken together, haplotypes within subgroups M2 and M3 might have originated from their core haplotypes as a result of domestication events followed by subsequent expansion.

The subgroup M2 is mainly distributed in the SEA region (Figure 2*D*). Of the 13 haplotypes representing 99 domestic pigs and eight wild boars in this subgroup, 10 haplotypes that accounted for 62 domestic pigs and one haplotype for four wild boars were found in this region. A small number of individuals were also found in such adjacent regions as Yunnan and ISEA (Figures 2*C* and *2B*). This result would support for a SEA origin of the subgroup M2.

The subgroup M3 is mainly found in the ISEA region (Figure 2*B*). Of the 23 haplotypes identified in 69 domestic pigs and 24 wild boars in this subgroup, 16 haplotypes representing 49 domestic pigs and seven wild boars were found in the ISEA area. In addition, two domestic haplotypes harboring 20 individuals were found in the Pacific Islands (Figure 2*A*), and 11 wild boar haplotypes were found in the areas of SEA and Yunnan (Figures 2*D* and *2C*). Thus the pattern is consistent with an ISEA origin of the subgroup M3.

The subgroup M1 is distributed across Asia and the Pacific Islands, and harbors 78 domestic haplotypes (representing 1,329 samples) and 34 wild haplotypes (representing 159 samples), between which 11 haplotypes are shared (Table S2). Obviously, subgroup M1 is the major subgroup of the haplogroup M. However, it is difficult to identify any potential domestical center(s) of this subgroup without the analysis of haplotype distributions among the 15 regions.

### The divisity gradient analysis of the subgroup M1 between different geographical regions

**Figure 3:**
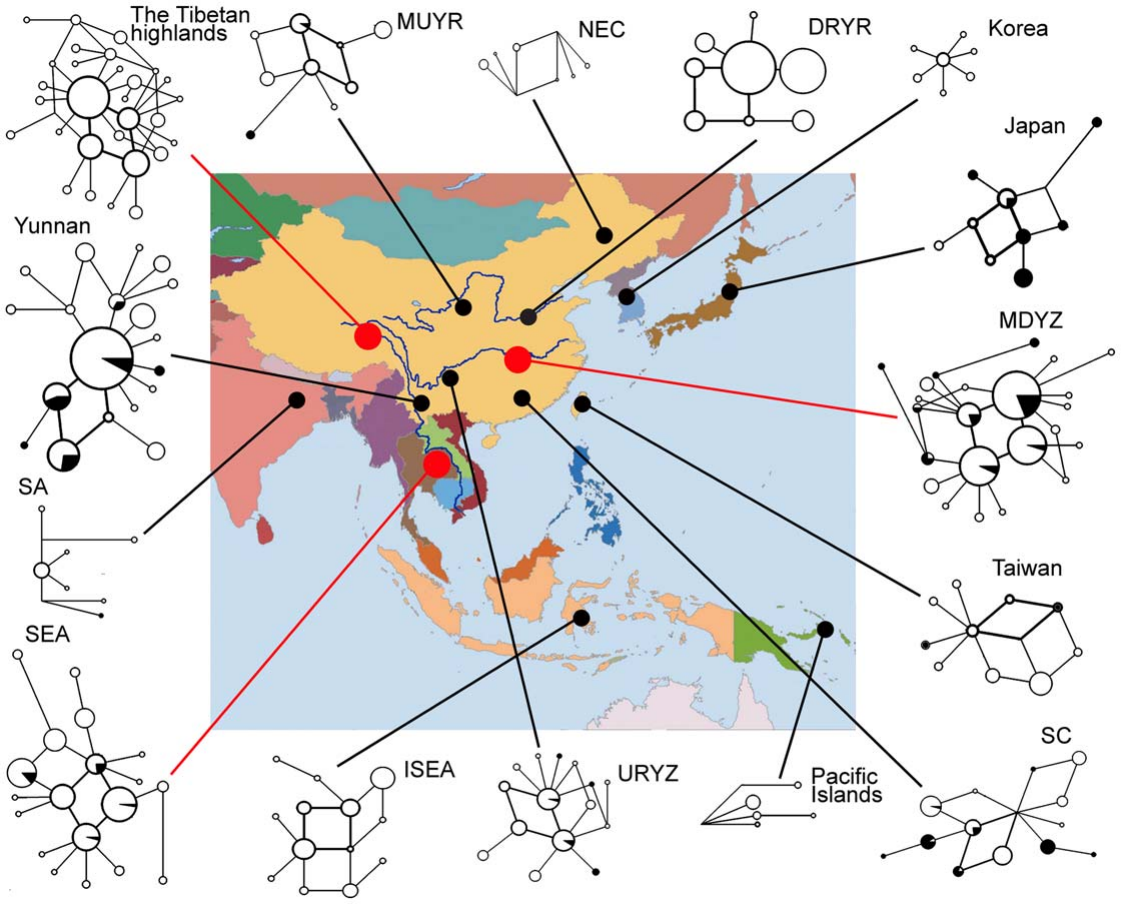
Median joining networks of the M1 subgroup. The haplotypes are symbolized by circles and are separated by one substitutional step. The length of each branch is proportional to the number of associated mutations. The four core haplotypes are indicated with bold lines. The size of the circle is proportional to the frequency of the haplotype and the white and black areas of the circle represent the proportion of this frequency made up by domestic pigs and wild boars. Three large red dots represent the three diversity centers of the Tibetan highlands, Southeastern Asia (SEA) and the middle and down stream region of the Yangtze River (MDYZ). The other 12 regions exhibited diversities that decreased along a gradient (see Table 2 for regional diversity). The abbreviations for the regions are explained in the main text.

The subgroup M1 possesses four widely distributed core haplotypes (Hap5, Hap7, Hap8 and Hap9; Table S2), which cover all of Asia and are represented by 820 domestic pigs and 76 wild boars in the current study. The haplotype network (Figure 3) highlights that the four core haplotypes form a parallelogram of one-mutation length, and possess considerable one, two, or greater than two mutation distance derivatives detectable in both domestic pigs and wild boars. The full extent of the diversity in the four core haplotypes within the subgroup M1 is best shown in regions such as MUYR, DRYR, URYZ, MDYZ, SEA, Yunnan, ISEA, Japan and the Tibetan highlands. However, the number of derived or unique haplotypes is the greatest in the SEA, Yunnan, MDYZ and Tibetan highland areas among these nine regions (Figure 3 and Table 2). If we presumably regard the Tibetan highlands, MDYZ and SEA regions as diversity centers, and Yunnan as their crossroads, the diversity of the M1 subgroup would then show a gradual decrease when moving outwards from these centers, whereas it could present a high value in Yunnan. Wild boars and domestic pigs across Asia share this subgroup and possess a roughly similar diversity gradient. Hereby, the result demonstrates that the domestication of the M1 subgroup could mainly occur in the three regions.

**Table 2:** Regional distributions of core haplotypes, derived haplotypes and unique haplotypes of domestic pigs and wild boars within subgroup M1.

Region	Domestic pig				Wild boar			
	Nc (N)	N1 (N)	N2 (N)	Nu (N)	Nc (N)	N1 (N)	N2 (N)	Nu (N)
Tibet	4 (122)	16 (62)	6 (14)	9 (20)	-	-	-	-
Yunnan	4 (110)	8 (33)	6 (21)	4 (20)	3 (20)	3 (5)	0	0
SEA	4 (100)	12 (69)	3 (8)	6 (27)	3 (5)	1 (4)	0	0
ISEA	4 (28)	7 (27)	2 (2)	4 (5)	0	0	0	0
Pacific	1 (1)	0	4 (16)	1 (10)	-	-	-	-
SA	1 (14)	2 (2)	3 (5)	3 (4)	0	0	1 (1)	1 (1)
URYZ	4 (75)	7 (28)	1 (1)	1 (2)	2 (3)	3 (4)	0	2 (3)
MDYZ	4 (190)	15 (36)	1 (1)	8 (9)	4 (25)	3 (4)	2 (3)	2 (3)
SC	3 (46)	4 (42)	1 (10)	2 (2)	2 (7)	5 (24)	2 (2)	2 (2)
Taiwan	2 (3)	5 (15)	0	5 (15)	1 (2)	1 (2)	0	0
MUYR	4 (28)	4 (20)	0	1 (9)	1 (1)	0	1 (2)	1 (2)
DRYR	4 (85)	4 (70)	0	1 (1)	-	-	-	-
NEC	2 (8)	1 (18)	0	0	1 (11)	4 (23)	0	1 (6)
Japan	4 (5)	1 (1)	0	0	2 (3)	3 (6)	1 (1)	0
Korea	1 (5)	6 (8)	0	6 (8)	0	3 (3)	0	0

Nc, number of core haplotypes; N1, number of one mutation distance derivatives; N2, number of two or greater than two mutation distance derivatives; Nu, number of unique haplotypes; Number of individuals is stated in parentheses; A short line denotes that no sample was collected.

## Discussion

### Small scale pig domestication episodes in Asia

At least six independent lineages are highlighted by the global pattern of mtDNA phylogeography of wild boars and domestic pigs [14]. The present study comprehensively analysing previously reported and novel mtDNA data provides valuable insights into the origins of Asian pigs and the associated small scale domestication episodes.

In Eastern Asia, haplogroups D2, D3 and D8 are dispersed across the Yangtze and Yellow River regions, suggesting that sporadic domestication episodes occurred in these two valley civilizations. Whereas the haplogroup D1 is an independent clade of Tibetan pigs from the highlands in the upstream region of the Yellow River and harbors 15 unique haplotypes. Evidence from ancient DNA also proves that a domestication episode appeared in this area [24]. Additionally, haplogroup DW2 was shared between one Taiwanese domestic pig and one Taiwanese wild boar, suggesting that wild introgression may have occurred.

Phylogenetic analysis of mtDNA haplotypes using domestic pigs and wild boars in the SEA region (Table S1) revealed three haplogroups DW3, D5 and D7, whilst the network analysis identified the subgroup M2. Notably, in this study we described the haplogroups D7 and DW3 for the first time. The haplogroup D5 and the subgroup M2 are identical to haplogoup MTSEA and are present in relatively high frequencies in domestic pigs in the mountainous areas of Cambodia and Laos [19]. Unexpectedly, the haplogroup D7 is mainly found in Myanmar and Thailand, whereas DW3 is shared by domestic pigs and wild boars within the borders of Thailand itself. In addition, the three haplogroups and subgroup M2 are restricted to SEA, indicating small scale domestication episodes in this region.

ISEA sequences recently submitted to GenBank (Table S1) were analyzed in this study. The subgroup M3 revealed by network analysis (Figure 2) and the divergent haplogroup D6 identified by phylogenetic analysis (Figure S1) are mainly restricted to this region. Importantly, the core haplotype Hap1 of the subgroup M3 was shared by four Indonesian wild boars and 26 native domestic pigs, notably, three further Indonesian wild boars possessed sequences that were only one- or two-mutation distance derivatives from Hap1. The subgroup M3 and haplogroup D6 were very similar to the Pacific clade described by Larson et al [14]. This result suggests that ISEA, but not SEA, was the center of domestication of the so-called Pacific clade (M3 and D6 here), although a total of 17 wild boars from the SEA region and Yunnan Province also fell into this subgroup. Thus, the present mtDNA evidence does not support previous results unfortunately that the Pacific clade was recently domesticated within SEA and subsequently dispersed throughout ISEA and Pacific Islands [15], [17], [24].

However, DW1 is an independent haplogroup located in SA and shared between domestic pigs and wild boars, which is also consistent with clade D3 as reported by Larson et al. [14]. Moreover, DW1 was found mainly in sympatric domestic and wild pigs in Northern India, suggesting that an independent domestication event occurred in the Indus Valley Civilization [14], [25]. In addition, haplogroup D4 was found only in domestic pigs in Bhutan, suggesting that a small scale domestication event occurred in the Himalayan foothills.

With the exception of haplogroup E, these 19 minor mtDNA lineages may correspond to distinct small scale domestication events, although the possibility of wild introgression cannot be excluded for those clades represented by only a few individuals.

### Domestication center of major subgroup M1 in Asia

The major subgroup M1 harbors nearly half of the haplotypes identified in this study (domestic pigs, 72/162; wild boars, 39/85) and over half of the individuals (domestic pigs, 1329/1663; wild boars, 159/292) (Table S1). This subgroup was represented by samples taken from a range of breeds in all the 15 examined regions. There was considerable overlap in the haplotype status of domestic pig samples from SEA, SC, URYZ, MDYZ, MUYR, Yunnan and Japan, in addition to associations with local wild boars. It is therefore difficult to specify any potential domestication site for subgroup M1 based on limited haplotype distribution or the shared status of domestic pigs and wild boars. The diversity gradient [26] judged by the number of derived haplotypes, the number of unique haplotypes and the proportionate allocation to the four core haplotypes, may have value in furthering our understanding of the origin of this subgroup. The present mtDNA data show a gradient in the diversity of subgroup M1 from high values within the diversity centers (MDYZ, SEA and the Tibetan highlands) to low values in the other regions (Figure 3, Table 2). These three areas may therefore be the site of origin. Subgroup M1 actually contained clades D2 (Eastern Asia) and D5 (Southeastern Asia) and haplogroups D1a2 (Yangtze River), D1b and D1a1a (Mekong region) found previously by the fine grained mtDNA phylogenomic analysis of wild boars and domestic pigs [14], [18]. However, the Tibetan highlands were revealed to be a potential diversity center and origin for the Tibetan pig for the first time in the present study. Great diversity is found in Yunnan which lies at the crossroads between the populations from the three different domestication centers of MDYZ, SEA and Tibet. The Yellow River, URYZ, NEC, SC, ISEA, and Pacific Islands regions, together with Japan, Korea and Taiwan, represent dispersal areas for the initial diversification. The gradual decrease in the diversity from SEA through ISEA and into the Pacific Islands suggests that subgroup M1 pigs in the Pacific Islands were probably domesticated in SEA and subsequently migrated through ISEA and into the Pacific Islands due to human colonization of remote Oceania associated with the Lapita cultural complex. This conclusion was reached without reference to the Pacific clade described by Lum et al [17] and Larson et al [15], [24].

### The origin of the Tibetan pig

Fifteen haplotypes (representing 17 individuals) of Tibetan pigs were found in the highly divergent D1 lineage, whilst the other Tibetan pigs belonged to subgroup M1 with the exception of one individual in group D3 and one in D7. The high divergence of the D1 haplogroup and association with one of the diversity centers of subgroup M1 provide two lines of evidence strongly suggesting that Tibetan pigs originated in the Tibetan highlands. Modern Tibetans are thought to be descendants of people who have occupied the Tibetan Plateau since the mid-Holocene (between 7,000 and 5,000 years ago) [27], or potentially even since the late Pleistocene (approximately 21,000 years ago) [28], [29]. 5000 years old domestic pig bones from the Kanuo archaeological site also suggest that Tibetan people domesticated the Tibetan pig [3]. All the evidence strongly support our hypothesis that the Tibetan pig has a local rigin.

Understandably, in most cases, the distribution of domestic animals may be expected to follow the migration of their owners. Previous genetic studies using maternal, paternal and autosomal markers suggest three separate origins of Tibetan people [29]–[34], namely Northern Mongolia, the Yellow River region and Central Asia. The Tibetan plateau is the largest and most geographically isolated area with a high altitude on earth and gradually descends to a low altitude only at its northeastern edge. The Tangbo Ancient Road was a highly important trade route snaking westward from Chang'an, and then across the Tanggula Mountains before reaching Lhasa. Unfortunately, no molecular data are now available to prove the three regions listed above as centers of origin of domestic pigs. Although ancient DNA samples from the genus *Sus* found from the Yellow River drainage basin also belonged to the most common haplotypes found throughout Asia [24], this could not be used to support the occurrence of a major domestication event in this region. It is suggested that only limited human-mediated dispersal of domestic pigs occurred from the three regions listed above into the Tibetan highlands via the Tangbo Ancient Road. Obviously, the Himalayan mountain range extends from Pakistan in the west to Burma in the east and forms a natural barrier between the Tibetan plateau and the Indian subcontinent. In addition, the diversity of SA region is very low. The genetic contribution from SA to the Tibetan pig may have been highly limited by this natural barrier. Meanwhile, the trade between Tibet and southwestern China was carried out through so called the Tea-horse Ancient Road including the Yunnan-Tibet and the Sichuan-Tibet routes, which together stretch across more than 4,000 kilometers of the Hengduan mountains. A genetic contribution to the Tibetan pig may still have occurred from SEA and the Yangtze River region but, in view of the extreme geographical isolation of the Tibetan highlands, this gene flow from the lowlands cannot readily explain the unexpectedly high diversity in the Tibetan highlands.

In conclusions, our results show that both the substantial genetic diversity and the highly divergent lineage present in the geographically isolated Tibetan highlands strongly support the local domestication of the Tibetan pig. Additionally, it may also be interesting to study the origins of other domestic animals indigenous to the Tibetan highlands in order to further confirm the conclusion.

## Supporting Information

Figure S1 **Bayesian consensus tree of 229 pig mtDNA control region haplotypes.** The haplotypes from Hap1 to Hap229 were found in 241 individuals sequenced in this study and 1714 reported sequences (Table S1). Haplotypes existing only in domestic populations, only in wild populations or existing in both were identified. The methods used to generate the tree are discussed in the main text. The digits at the nodes are posterior probabilities. The regional distributions of haplotypes and haplotype sharing between domestic pigs and wild boars within the same haplogroups are presented in Tables S2 and 1.
(TIF)

Table S1 **Information on sampling of Asian domestic pigs and wild boar.**
(XLS)

Table S2 **Haplotype and geographical distributions across 15 regions.**
(XLS)
