# Supplementary figures and images for: The Local Origin of the Tibetan Pig and Additional Insights into the Origin of Asian Pigs
Source: PLoS One. 2011 Dec 7;6(12):e28215. doi: 10.1371/journal.pone.0028215 (PMC3233571; doi:10.1371/journal.pone.0028215)

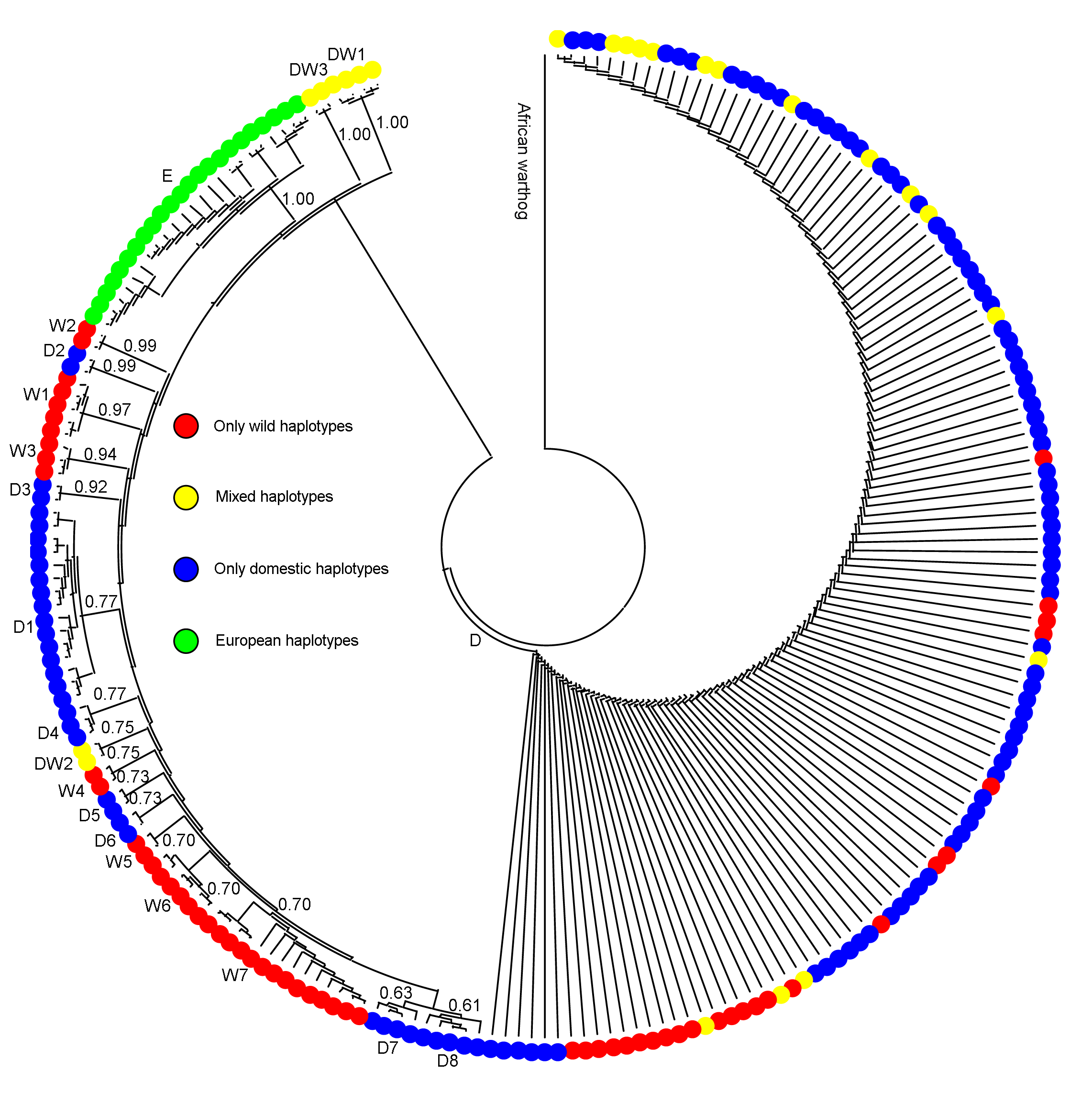

Supplement: Figure S1 — Bayesian consensus tree of 229 pig mtDNA control region haplotypes. The haplotypes from Hap1 to Hap229 were found in 241 individuals sequenced in this study and 1714 reported sequences (Table S1). Haplotypes existing only in domestic populations, only in wild populations or existing in both were identified. The methods used to generate the tree are discussed in the main text. The digits at the nodes are posterior probabilities. The regional distributions of haplotypes and haplotype sharing between domestic pigs and wild boars within the same haplogroups are presented in Tables S2 and 1. (TIF) [file pone.0028215.s001.tif]
